# FDG uptake is a surrogate marker for defining the optimal biological dose of the mTOR inhibitor everolimus *in vivo*

**DOI:** 10.1038/sj.bjc.6605076

**Published:** 2009-05-12

**Authors:** D Cejka, C Kuntner, M Preusser, M Fritzer-Szekeres, B J Fueger, S Strommer, J Werzowa, T Fuereder, T Wanek, M Zsebedics, M Mueller, O Langer, V Wacheck

**Affiliations:** 1 Department of Clinical Pharmacology, Medical University of Vienna, Vienna, Austria; 2 Department of Radiopharmaceuticals and microPET Imaging, Austrian Research Centers GmbH-ARC, Seibersdorf, Austria; 3 Division of Oncology, Department of Internal Medicine I, Medical University of Vienna, Vienna, Austria; 4 Clinical Institute of Medical and Chemical Laboratory Diagnostics, Medical University of Vienna, Vienna, Austria; 5 Department of Radiology, Medical University of Vienna, Vienna, Austria; 6 Department of Toxicology, Austrian Research Centers GmbH-ARC, Seibersdorf, Austria

**Keywords:** PK/PD, rapamycin, dose finding

## Abstract

This study aimed to test whether [^18^F]fluoro-D-glucose (FDG) uptake of tumours measured by positron emission tomography (PET) can be used as surrogate marker to define the optimal biological dose (OBD) of mTOR inhibitors *in vivo*. Everolimus at 0.05, 0.5, 5 and 15 mg kg^−1^ per day was administered to gastric cancer xenograft-bearing mice for 23 days and FDG uptake of tumours was measured using PET from day 1 to day 8. To provide standard comparators for FDG uptake, tumour volume, S6 protein phosphorylation, Ki-67 staining and everolimus blood levels were evaluated. Everolimus blood levels increased in a dose-dependent manner but antitumour activity of everolimus reached a plateau at doses ⩾5 mg kg^−1^ per day (tumour volume treated *vs* control (T/C): 51% for 5 mg kg^−1^ per day and 57% for 15 mg kg^−1^ per day). Correspondingly, doses ⩾5 mg kg^−1^ per day led to a significant reduction in FDG uptake of tumours. Dose escalation above 5 mg kg^−1^ per day did not reduce FDG uptake any further (FDG uptake T/C: 49% for 5 mg kg^−1^ per day and 52% for 15 mg kg^−1^ per day). Differences in S6 protein phosphorylation and Ki-67 index reflected tumour volume and changes in FDG uptake but did not reach statistical significance. In conclusion, FDG uptake might serve as a surrogate marker for dose finding studies for mTOR inhibitors in (pre)clinical trials.

Mammalian target of rapamycin (mTOR) inhibitors such as everolimus are under intensive investigation for cancer therapy. In 2007, the rapamycin derivative temsirolimus was FDA approved for the treatment of renal cell cancer (Hudes *et al*, 2007).

Dose escalation of mTOR inhibitors beyond a certain level does not improve antitumour activity but may increase the incidence of adverse effects (Atkins *et al*, 2004; Chan *et al*, 2005; Tabernero *et al*, 2008). Therefore, an optimal biological dose (OBD) seems to exist for mTOR inhibitors. This is in contrast to the concept of maximum tolerated dose (MTD) commonly used for conventional cytotoxic therapy. However, determination of OBD for mTOR inhibitors is still elusive because there is currently no standard surrogate marker available for translating preclinical research results into clinical practice. In previous studies the phosphorylation status of mTOR targets such as S6 protein from surrogate tissue or tumour samples has been commonly used as a marker of mTOR kinase activity and the degree of mTOR inhibition (Boulay *et al*, 2004; Duran *et al*, 2006). Similarly, decreases of the proliferation marker Ki-67 in tumour samples have been correlated with mTOR inhibitor activity (Neshat *et al*, 2001; Tabernero *et al*, 2008). These biomarker approaches clearly have their limitations if it comes to clinical testing because the serial biopsies required for such measurements are rarely feasible. Hence, new strategies to determine the OBD of mTOR inhibitors are needed.

Changes in tumour metabolism caused by targeted therapeutics have been successfully studied using 2′-deoxy-2′-[^18^F]fluoro-D-glucose and positron emission tomography (FDG-PET; Stroobants *et al*, 2003; Chen *et al*, 2007). It has been shown that decreases in tumour FDG uptake can be seen as early as 24 h after administration of a single dose of temsirolimus in a murine model of renal cell cancer (Thomas *et al*, 2006). Furthermore, FDG-PET has been used to distinguish between tumours, which are sensitive or resistant to mTOR inhibitors in various preclinical cancer models (McSheehy *et al*, 2005; Wei *et al*, 2008). So far, only the effects of relatively high doses of mTOR inhibitors on tumour FDG uptake have been studied. However, guidance of dose finding studies of mTOR inhibitors using a non-invasive method such as PET would be highly attractive, especially in clinical settings.

In this study, we have set out to test whether quantitative changes in tumour FDG uptake qualify as a surrogate endpoint for predicting the dose-dependent antitumour activity of the mTOR inhibitor everolimus.

## Materials and methods

### Gastric cancer cells and compound

The NCI-N87 human gastric cancer cell line (intestinal type) was purchased from ATCC (Manassas, VA, USA). Everolimus was kindly provided by The Novartis Institutes for BioMedical Research Basel, Oncology, Switzerland. Formulated everolimus was supplied as oral microemulsion. Aliquots were stored at −20°C and diluted with 5% glucose solution just before administration. Microemulsion without everolimus was provided as placebo. Everolimus was measured in EDTA-whole blood by an integrated online solid-phase extraction high-performance liquid chromatography single-quad mass spectrometry (SPE-HPLC-MS) system (Kirchner *et al*, 2001). The detection limit for everolimus was 3 ng ml^−1^.

### Western blot

Snap-frozen tumour xenografts were pulverised with a MM 200 mixer mill (Retsch, Haan, Germany) and protein was extracted with lysis buffer as described previously (Cejka *et al*, 2008). 10 *μ*g protein per lane was loaded on a 10% SDS–polyacrylamide gel and separated by electrophoresis followed by blotting on nitrocellulose membranes (Schleicher & Schuell, Dassel, Germany). Membranes were blocked with I-Block (Tropix, Bedford, MA, USA) in PBS with 0.1% Tween blocking solution at room temperature for 1 h followed by incubation with primary antibodies diluted in blocking solution at 4°C over night. Antibodies were directed against phospho-S6 Ser240/244 (Cell Signaling Technology, Danvers, MA, USA) or tubulin (Sigma Aldrich, Vienna, Austria). Primary antibodies were detected by anti-rabbit alkaline phosphatase-conjugated secondary antibodies (Tropix; 1 : 5000) and visualised by chemiluminescence using CSPD (Tropix) substrate.

### Tumour xenograft model

Pathogen-free, 4- to 6-week old, female athymic nude mice (Harlan-Winkelmann, Borchen, Germany) were housed under sterile conditions and treated according to the regulations imposed and approved by the local animal welfare committee. Mice were inoculated by s.c. injection into both upper flanks with 100 *μ*l of a tumour cell suspension in DPBS containing 10^7^ NCI-N87 cells. Tumour volume was measured twice weekly using a calliper and calculated according to the approximation formula: volume (mm^3^)=*π*/6 × (*L* × *W*^2^), where *L* represents length defined as the largest tumour extension in the coronary plane and *W* represents the width defined as the largest tumour extension perpendicular to *L* in the same plane.

The N87 gastric cancer xenograft nude mouse model was used because it has previously been found to be sensitive to mTOR inhibition at a dose of 5 mg kg^−1^ per day everolimus (Cejka *et al*, 2008).

In the first part of the study, mice (*n*=5 per group) were randomised to receive 200 *μ*l of microemulsion placebo or 5 mg kg^−1^ per day everolimus p.o. 5 × per week. Therapy was initiated 10 days after tumour inoculation and mice were killed 16 days after initiation of therapy.

In the second part of the study, mice (*n*=4 per group) were assigned randomly to one of the following treatment groups 10 days after xenografting:

1. microemulsion placebo p.o. 5 × per week
2. everolimus 0.05 mg kg^−1^ per day p.o. 5 × per week
3. everolimus 0.5 mg kg^−1^ per day p.o. 5 × per week
4. everolimus 5 mg kg^−1^ per day p.o. 5 × per week
5. everolimus 15 mg kg^−1^ per day p.o. 5 × per week

Mice were treated for 23 days.

In both experiments, microPET measurements preceded gavaging of mice.

### microPET

Synthesis and quality control of FDG was performed by using standard methods (Hamacher *et al*, 1986). PET scans were acquired using the microPET Focus 220 system (Siemens Medical Solutions USA Inc., Knoxville, TN, USA) as described previously (Tai *et al*, 2005). As animal handling has been found to have a considerable impact on FDG-PET results, it was standardised for all animals (Lee *et al*, 2005; Fueger *et al*, 2006). In brief, mice were deprived of food for 6–8 h before FDG injection. Mice had access to drinking water at all times. Body warming was achieved by placing the entire cage on a heating pad kept at 38°C. Warming was started around 30 min before tracer injection and continued throughout the uptake and imaging period. FDG (7.4 MBq in a volume of 100 *μ*l of physiological saline) was administered by tail vein injection under isoflurane anaesthesia (2%). Mice were kept under isoflurane anaesthesia throughout the whole uptake and imaging period. A 10 min static image (250–750 keV energy window, 6 ns timing window) was acquired at 1 h after FDG injection. Baseline imaging was performed before initiation of treatment (day 0). Imaging was repeated on days 1, 2 and 8 (and additionally on day 15 in the first part of the study) just before administration of everolimus with identical acquisition parameters. Images were reconstructed using FORE rebinning followed by filtered back projection algorithm resulting in a voxel size of 0.4 × 0.4 × 0.796 mm^3^. The standard data correction protocol (normalisation, decay correction and injection decay correction) was applied to the data.

A calibration factor for converting units of microPET images into absolute radioactivity concentration units was first generated by imaging a phantom filled with a known concentration of FDG. Radioactivity concentration in tumours was quantified in terms of percent injected dose per gram tissue (%ID g^−1^) using the image analysis software Amide (Loening and Gambhir, 2003). Volumes of interest (VOIs) were manually drawn around the whole two tumours on the reconstructed images. A background region in the abdomen was also defined. Radioactivity uptake in tumour tissue was normalised to that of normal tissue and expressed as tumour-to-background (T/B) ratio, whereby only those pixels exhibiting >75% of maximum radioactivity uptake in the tumour VOI were included.

### Immunohistochemistry

For quantification of tumour cell proliferation, Ki-67 antigen was detected immunohistochemically. Specimens were fixed in 4.5% phosphate-buffered formaldehyde solution, embedded in paraffin and cut at a thickness of 3–5 *μ*m. Sections were deparaffinised in xylol. Slides were heated in 0.01 M citrate buffer (pH 6.0) for 20 min (for anti-Ki-67 immunostaining) in a microwave oven at 600 W. Anti-Ki-67 (monoclonal rabbit; Thermo Fisher Scientific, Fremont, CA, USA; dilution 1 : 200) was used to detect tumour cell proliferation. Detection of immunostaining was performed using a biotinylated anti-rabbit secondary antibody (Vector Laboratories, Burlingame, CA, USA), an avidin/biotin blocking kit (Vector Laboratories) and DAB as chromogen (Vector Laboratories). For quantitative assessment of Ki-67 immunolabelling, a total of 500 tumour cells were evaluated in each specimen in fields showing the highest density of immunopositive cells. The fraction of labelled tumour cells was expressed as percentage (proliferation index).

### Statistical analysis

Data is presented as mean±s.e.m. unless indicated otherwise. The ratio between treated and control groups is presented as % T/C where appropriate. To illustrate the relationship between FDG uptake and everolimus dose, T/B ratios of single tumours were inverted and the mean T/B ratio of the placebo group was subtracted from these values. Accordingly, a value of 0 represents no difference in FDG uptake compared to placebo. The highest dose group was defined as showing the maximum difference in FDG uptake compared to placebo, which was set to a value of 1 (arbitrary units). A sigmoidal dose–response curve was fitted to the data using the Hill equation. To test for statistical significance of changes within treatment groups, data were analysed using the Wilcoxon signed-ranks test. For multiple comparisons, analysis of variance (ANOVA) was followed by Tamhane's T2 *post hoc* test. Correlations between two parameters were studied using the two-tailed Spearman's rho-test. *P* values <0.05 were considered statistically significant. Statistical analysis was performed using SPSS 13 software (SPSS Inc., Chicago, IL, USA).

## Results

**Figure 1:**
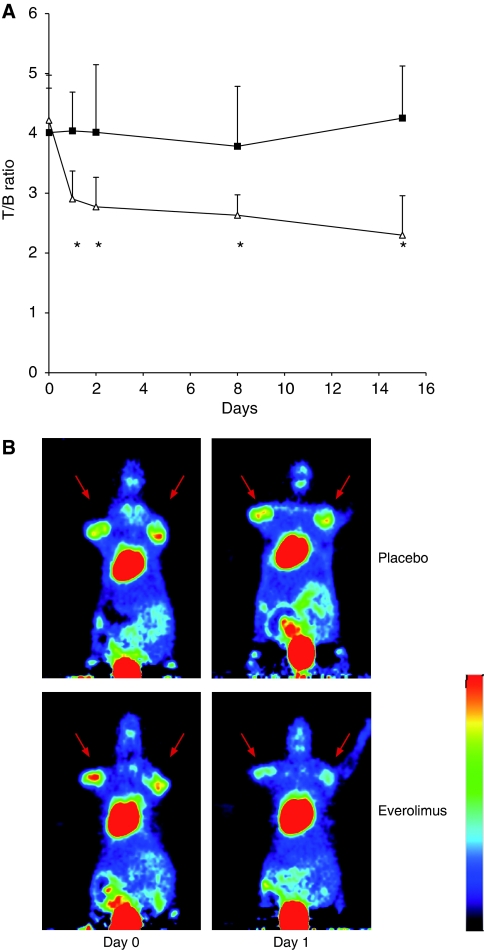
(**A**) Tumour-to-background ratio of FDG uptake of xenograft-bearing mice treated with placebo (solid squares) or 5 mg kg^−1^ per day everolimus (open triangles). Asterisks indicate statistically significant differences in tracer uptake between everolimus-treated tumours and control. (**B**) Representative FDG-PET scans. N87 gastric cancer cells were inoculated bilaterally into the upper flanks of nude mice. Xenograft tumours (arrows) show decreases in tracer uptake early after initiation of everolimus treatment, whereas FDG uptake in the control group remains stable. The radiation scale was set from 0 to 7 %ID g^−1^.

In a pilot study, we first investigated whether FDG uptake is influenced by everolimus in the NCI-N87 gastric cancer xenograft model. As shown in Figure 1, treatment with everolimus at a dose of 5 mg kg^−1^ per day resulted in a rapid and significant decline of FDG uptake beginning 24 h after initiation of treatment (day 1: a reduction of 31% relative to baseline, *P*<0.001). This significant decrease of FDG uptake was observed at all time points measured (day 15: −45%; *P*<0.001). In contrast, in the sham-treated control group FDG uptake did not show any significant change throughout the study (mean change from baseline 0.3±3%). FDG uptake in the everolimus group was significantly lower than in the control group at any studied point in time (71% T/C on day 1 to 54% TC on day 15, *P*<0.01 for all). During the course of this experiment, tumour volume increased from 306 to 1046 mm^3^ in the placebo group and from 258 to 408 mm^3^ in the everolimus group, which indicates that the reduction in tracer uptake in everolimus-treated tumours was not due to tumour shrinkage.

**Figure 2:**
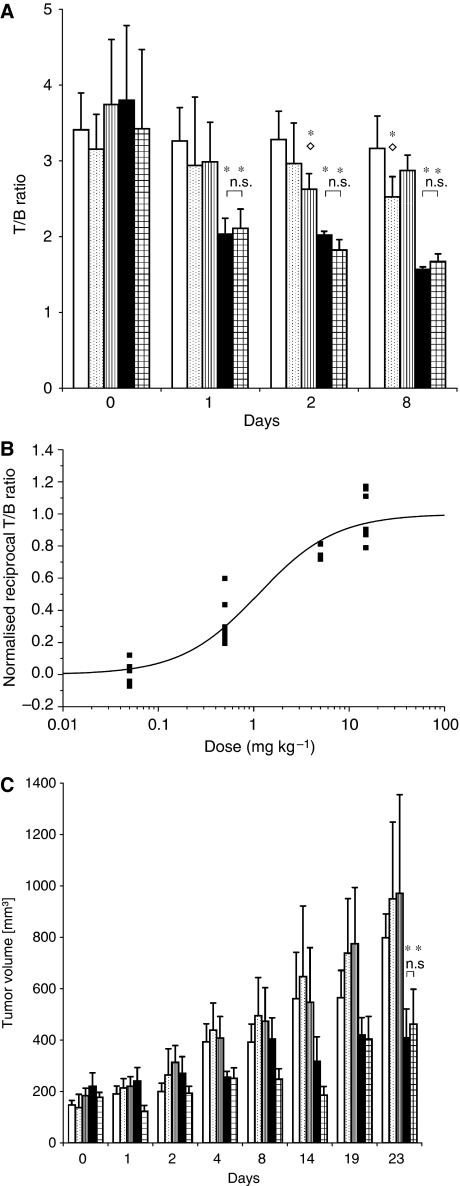
(**A**) Tumour-to-background ratio of FDG uptake of xenograft tumours on different days after initiation of treatment with placebo (open bars) or everolimus at 0.05 mg kg^−1^ per day (dotted bars), 0.5 mg kg^−1^ per day (vertical lines), 5 mg kg^−1^ per day (solid black) or 15 mg kg^−1^ per day (chequered). Asterisks indicate statistically significant differences in tracer uptake compared to placebo, 0.05 and 0.5 mg kg^−1^ per day everolimus. Open diamonds indicate statistically significant differences in tracer uptake compared to 5 and 15 mg kg^−1^ per day everolimus; n.s. indicates no statistically significant differences of FDG uptake between 5 and 15 mg kg^−1^ per day everolimus. (**B**) Relationship of FDG uptake of tumours based on T/B ratio and everolimus dose 48 h after initiation of treatment. A normalised reciprocal T/B ratio of 0 indicates no difference in FDG uptake compared to placebo. A normalised reciprocal T/B ratio of 1 is defined as maximum difference in tracer uptake between placebo and everolimus-treated tumours. Solid squares represent individual tumours. Everolimus dose is given on a logarithmic scale. (**C**) Tumour volume of subcutaneous xenografts derived from manual calliper measurements. Asterisks indicate statistically significant differences in tumour volume compared to placebo, 0.05 and 0.5 mg kg^−1^ per day everolimus; n.s. indicates no statistically significant differences in tumour volume between 5 and 15 mg kg^−1^ per day everolimus.

Based on these findings, we then studied the dose–effect relationship between everolimus and FDG uptake in NCI-N87 xenografts. Everolimus treatment resulted in a dose-dependent decrease in tumour FDG uptake (Figure 2A). At all studied points in time everolimus at doses of ⩾5 mg kg^−1^ led to a significant decrease in FDG uptake compared to placebo. Tumour FDG uptake ranged from 62% T/C on day 1 to 49% T/C on day 8 for the 5 mg kg^−1^ per day group (*P*<0.001, all measurements). FDG uptake did not differ between the 5 mg kg^−1^ per day and the 15 mg kg^−1^ per day group throughout the study (*P*>0.05, all measurements). FDG uptake in the 0.05 and 0.5 mg kg^−1^ per day groups was only marginally lower than in the placebo group (range: 93–75% T/C). With the exception of the 0.05 mg kg^−1^ per day group on day 1, FDG tumour uptake of groups treated with everolimus <5 mg kg^−1^ per day was significantly higher than in groups ⩾5 mg kg^−1^ per day at all studied points in time (*P*<0.017 to *P*<0.001). A dose–effect curve showing the relationship between everolimus dose and FDG uptake is shown in Figure 2B.

To correlate FDG-PET results with tumour volumes, cancer xenografts were measured manually with a calliper (Figure 2C). After establishment of palpable tumours (mean tumour volume: 173±43 mm^3^), mice were randomised to receive placebo or everolimus treatment (0.05, 0.5, 5 and 15 mg kg^−1^ per day, respectively). Starting tumour volumes did not differ significantly between groups. At the end of the experiment, differences in tumour volumes between placebo (797 mm^3^) and everolimus at doses of 0.05 mg kg^−1^ per day (949 mm^3^) and 0.5 mg kg^−1^ per day (969 mm^3^) were not statistically significant (*P*=n.s. for all). Only tumour volumes in the ⩾5 mg kg^−1^ per day groups differed significantly from placebo control (5 mg kg^−1^ per day: 408 mm^3^, 51% T/C, *P*<0.04; 15 mg kg per day: 462 mm^3^, 57% T/C, *P*<0.02). No statistically significant difference in tumour volume was observed between the 5 and 15 mg kg^−1^ per day groups (*P*=n.s.). Early changes in FDG uptake of tumours were found to correlate significantly with tumour size after long-term therapy at all three points in time studied by PET. There was a positive correlation between tumour volume at day 23 and FDG uptake, with correlation coefficients of 0.67, 0.68 and 0.61 for days 1, 2 and 8, respectively (*P*<0.001 for all).

**Figure 3:**
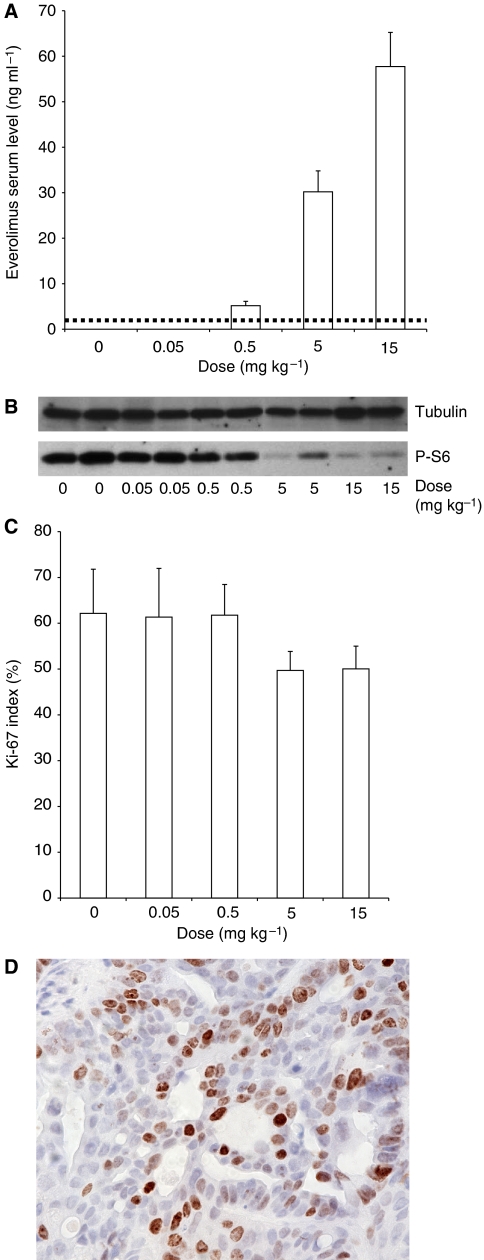
(**A**) Everolimus through-levels in whole blood of tumour bearing mice as measured by a SPE-HPLC-MS assay. Blood was drawn 24 h after the last administration of everolimus immediately before animal killing. Detection limit of the assay was 3 ng ml^−1^ (dashed line). (**B**) Phosphorylation status of S6 protein (Ser 240/244) from tumour tissue. Two tumour lysates from every dose group were randomly selected to study dose-dependent effects of increasing doses of everolimus on S6 phosphorylation by western blotting. Tubulin serves as loading control. (**C**) Proliferation index of gastric cancer cells *in vivo*. Quantitative assessment of proliferating tumour cells in relation to everolimus dose based on immunohistochemical staining of Ki-67 antigen of excised, formalin fixed, paraffin-embedded tumour tissue. (**D**) Representative immunohistochemical staining of Ki-67 positive cells.

To compare FDG-PET results with standard PK/PD biomarkers for mTOR inhibitors, everolimus blood levels and PD effects were studied. Everolimus trough levels were measured in whole blood drawn immediately before killing the animals (Figure 3A). A dose-dependent increase in everolimus blood levels beginning from the 0.5 mg kg^−1^ per day dose group up to the 15 mg kg^−1^ per day group was found. Everolimus was below the detection limit of the assay in the 0.05 mg kg^−1^ per day group.

Tumour xenografts were analysed for changes of S6 protein phosphorylation status by western blotting. As shown in Figure 3B, a dose-dependent decrease in S6 phosphorylation at Ser240/244 was observed. The most prominent decline in S6 protein phosphorylation was observed in the 5 and 15 mg kg^−1^ per day groups with no significant difference between both groups.

Changes in tumour cell proliferation elicited by everolimus treatment were assessed by immunohistochemical staining of Ki-67 antigen (Figure 3C and D). Although statistically not significant, there was a trend for lower tumour cell proliferation with higher everolimus dose. The proliferation index for both everolimus dose groups <5 mg kg^−1^ per day was similar to sham-treated control (62 and 61%, respectively) whereas proliferation index of the two everolimus dose groups ⩾5 mg kg^−1^ per day was lower (49 and 50% for 5 and 15 mg kg^−1^ per day, respectively).

## Discussion

In the past, dose finding for targeted therapeutics such as mTOR inhibitors was mainly based on the concept of MTD, an approach ignoring the possibility to define an OBD with a potentially improved risk/benefit ratio for the patient. The general appeal of the OBD concept is tempered by the fact that translational studies relying on PK/PD biomarkers from tumour tissue or surrogate tissues may suffer from significant drawbacks for guiding preclinical and clinical dose finding.

For the first time, this study shows that dose-dependent changes in tumour FDG uptake can be used to identify the OBD of an mTOR inhibitor *in vivo*. Dose escalation beyond 5 mg kg^−1^ per day does not result in enhanced antitumour effect of everolimus as determined by measuring tumour volumes. Therefore, 5 mg kg^−1^ per day everolimus is defined as the OBD in this tumour model. As early as 24 h after initiation of therapy, tumours of animals receiving the OBD of 5 mg kg^−1^ per day show a significant decrease in FDG uptake compared to placebo control. Additionally, tracer uptake in the OBD group differs significantly from lower dose groups after 48 h of therapy. Escalating everolimus up to 15 mg kg^−1^ per day does not reduce tracer uptake further throughout the study, although serum everolimus levels were markedly higher in the 15 mg kg^−1^ per day group than in the 5 mg kg^−1^ per day (OBD) group. Moreover, tumour growth is inhibited to a similar extent in the 5 mg kg^−1^ per day group as in the 15 mg kg^−1^ per day group. Hence, the tumour growth-inhibitory effect of various doses of everolimus is reflected by a decrease of tumour FDG uptake, which appears to function as a surrogate marker.

Changes in cellular signalling in blood cells or other surrogate tissues do not necessarily mirror changes in solid tumour cell signalling. Because of their invasive nature, serial tumour tissue biopsies will hardly be available in clinical trials. If so, the heterogeneity of tumour architecture in these biopsies will hamper evaluation of protein phosphorylation status. Similar considerations apply to immunohistochemical techniques or mTOR kinase activity assays. Analysis of protein phosphorylation is commonly performed by western blotting, a method with very limited usefulness for quantitative assessment. Furthermore, changes in phosphorylation of molecules downstream of mTOR do not necessarily correspond with tumour response (Wei *et al*, 2008).

mTOR inhibitors have been shown to posses antiangiogenic activity (Guba *et al*, 2002; Frost *et al*, 2004). However, the validity of angiogenesis markers such as microvascular density (MVD) to monitor antiangiogenic treatments is controversial (Hlatky *et al*, 2002). Treatment with everolimus can induce central tumour necrosis without any significant change in MVD whereas overall tumour volume continues to increase (Cejka *et al*, 2008). Therefore, clinical response criteria such as CT-scan-based RECIST (Therasse *et al*, 2000) or evaluation of treatment response by studying tumour vascularisation may not apply to mTOR-inhibitor therapy. It has been reported that there is a positive correlation between tumour blood flow and tumour FDG-uptake in breast cancer patients when both parameters are measured within minutes of tracer uptake (Zasadny *et al*, 2003). Therefore, decreases in FDG-uptake in everolimus-treated NCI-N87 xenografts may reflect changes in tumour perfusion instead of tumour cell proliferation. However, tracer uptake in this study was measured 1 h after administration of FDG, allowing for sufficient FDG extraction. Furthermore, tumour perfusion has been reported to be inversely correlated with PET-tracer uptake 1 h after administration of FDG in a tumour xenograft model (Pugachev *et al*, 2005), rendering perfusion-related bias in our study unlikely. Thus, reductions in FDG-uptake of NCI-N87 xenografts more likely result from downregulation of enzymes involved in cellular glucose metabolism such as hexokinase (Wei *et al*, 2008).

The validity of FDG-uptake as a surrogate marker for defining the OBD of everolimus is supported by our PD marker results commonly used for evaluating mTOR inhibition. Cancer cell proliferation measured by Ki-67 expression, as well as analysis of S6 phosphorylation status, also favours 5 mg kg^−1^ per day as OBD. Interestingly, effects of everolimus on S6 phosphorylation and cancer cell proliferation seem to be more binary when compared to PET results, which appear to be more graded. There is a clear decrease in S6 protein phoshporylation at everolimus doses at or above OBD compared to lower doses. Similarly, cancer cell proliferation is very similar between groups below OBD, whereas proliferation is lower in groups dosed at or above OBD. Again, there is little difference in cancer cell proliferation between groups when everolimus is dosed at OBD or higher. Results from tumour volume measurements also suggest more of a binary effect of everolimus treatment on tumour growth. In contrast to this, PET results suggest a more sigmoidal dose–effect relationship. One possible explanation would be that compared to other assays, PET is more sensitive in detection of small changes in tumour metabolism, which do not translate into attenuation of tumour growth. Alternatively, there might be a time delay between changes in S6 phosphorylation status, cancer cell proliferation and changes in FDG uptake. The relatively short half-life of everolimus may result in short-lived inhibition of mTOR signalling at low doses, which may be sufficient to alter FDG uptake, but insufficient to induce detectable differences in S6 protein phosphorylation, cancer cell proliferation and eventually tumour size. PET measurements were always done before treatment with everolimus, that is, at everolimus through-levels. At that time, S6 protein might have already recovered in the groups treated with low doses of everolimus, whereas effects on FDG uptake were still detectable by PET.

However, given the limitations of invasive biomarker sampling mentioned above, FDG-PET appears to be superior in defining the OBD of everolimus in this preclinical model. Furthermore, due to very good homogeneity of data generated with PET measurements compared to the other biomarker studies, statistically significant results can be generated with FDG-PET using relatively small sample sizes. Of note, PK data of everolimus blood levels provided no help for defining the OBD.

In conclusion, our findings support the concept that metabolic changes elicited by everolimus and visualised by PET are closely linked with the antitumour activity of the mTOR inhibitor everolimus. FDG uptake measured by PET can be used as a surrogate marker to determine the OBD of an mTOR inhibitor *in vivo* soon after initiation of therapy. Given the difficulties and limitations with other approaches to establish PK/PD relationships for mTOR inhibitors, FDG-PET may be a promising translational strategy to identify the OBD of mTOR inhibitors in clinical trials.

## References

[bib1] Atkins MB, Hidalgo M, Stadler WM, Logan TF, Dutcher JP, Hudes GR, Park Y, Liou SH, Marshall B, Boni JP, Dukart G, Sherman ML (2004) Randomized phase II study of multiple dose levels of CCI-779, a novel mammalian target of rapamycin kinase inhibitor, in patients with advanced refractory renal cell carcinoma. J Clin Oncol 22: 909–918 14990647 10.1200/JCO.2004.08.185

[bib2] Boulay A, Zumstein-Mecker S, Stephan C, Beuvink I, Zilbermann F, Haller R, Tobler S, Heusser C, O'Reilly T, Stolz B, Marti A, Thomas G, Lane HA (2004) Antitumour efficacy of intermittent treatment schedules with the rapamycin derivative RAD001 correlates with prolonged inactivation of ribosomal protein S6 kinase 1 in peripheral blood mononuclear cells. Cancer Res 64: 252–261 14729632 10.1158/0008-5472.can-3554-2

[bib3] Cejka D, Preusser M, Woehrer A, Sieghart W, Strommer S, Werzowa J, Fuereder T, Wacheck V (2008) Everolimus (RAD001) and anti-angiogenic cyclophosphamide show long-term control of gastric cancer growth *in vivo*. Cancer Biol Ther 7: 1377–1385 18708754 10.4161/cbt.7.9.6416

[bib4] Chan S, Scheulen ME, Johnston S, Mross K, Cardoso F, Dittrich C, Eiermann W, Hess D, Morant R, Semiglazov V, Borner M, Salzberg M, Ostapenko V, Illiger HJ, Behringer D, Bardy-Bouxin N, Boni J, Kong S, Cincotta M, Moore L (2005) Phase II study of temsirolimus (CCI-779), a novel inhibitor of mTOR, in heavily pretreated patients with locally advanced or metastatic breast cancer. J Clin Oncol 23: 5314–5322 15955899 10.1200/JCO.2005.66.130

[bib5] Chen W, Delaloye S, Silverman DH, Geist C, Czernin J, Sayre J, Satyamurthy N, Pope W, Lai A, Phelps ME, Cloughesy T (2007) Predicting treatment response of malignant gliomas to bevacizumab and irinotecan by imaging proliferation with [18F] fluorothymidine positron emission tomography: a pilot study. J Clin Oncol 25: 4714–4721 17947718 10.1200/JCO.2006.10.5825

[bib6] Duran I, Kortmansky J, Singh D, Hirte H, Kocha W, Goss G, Le L, Oza A, Nicklee T, Ho J, Birle D, Pond GR, Arboine D, Dancey J, viel-Ronen S, Tsao MS, Hedley D, Siu LL (2006) A phase II clinical and pharmacodynamic study of temsirolimus in advanced neuroendocrine carcinomas. Br J Cancer 95: 1148–1154 17031397 10.1038/sj.bjc.6603419 PMC2360568

[bib7] Frost P, Moatamed F, Hoang B, Shi Y, Gera J, Yan H, Frost P, Gibbons J, Lichtenstein A (2004) *In vivo* antitumour effects of the mTOR inhibitor CCI-779 against human multiple myeloma cells in a xenograft model. Blood 104: 4181–4187 15304393 10.1182/blood-2004-03-1153

[bib8] Fueger BJ, Czernin J, Hildebrandt I, Tran C, Halpern BS, Stout D, Phelps ME, Weber WA (2006) Impact of animal handling on the results of 18F-FDG PET studies in mice. J Nucl Med 47: 999–1006 16741310

[bib9] Guba M, von Breitenbuch P, Steinbauer M, Koehl G, Flegel S, Hornung M, Bruns CJ, Zuelke C, Farkas S, Anthuber M, Jauch KW, Geissler EK (2002) Rapamycin inhibits primary and metastatic tumour growth by antiangiogenesis: involvement of vascular endothelial growth factor. Nat Med 8: 128–135 11821896 10.1038/nm0202-128

[bib10] Hamacher K, Coenen HH, Stocklin G (1986) Efficient stereospecific synthesis of no-carrier-added 2-[18F]-fluoro-2-deoxy-D-glucose using aminopolyether supported nucleophilic substitution. J Nucl Med 27: 235–238 3712040

[bib11] Hlatky L, Hahnfeldt P, Folkman J (2002) Clinical application of antiangiogenic therapy: microvessel density, what it does and doesn't tell us. J Natl Cancer Inst 94: 883–893 12072542 10.1093/jnci/94.12.883

[bib12] Hudes G, Carducci M, Tomczak P, Dutcher J, Figlin R, Kapoor A, Staroslawska E, Sosman J, McDermott D, Bodrogi I, Kovacevic Z, Lesovoy V, Schmidt-Wolf IG, Barbarash O, Gokmen E, O'Toole T, Lustgarten S, Moore L, Motzer RJ (2007) Temsirolimus, interferon alfa, or both for advanced renal-cell carcinoma. N Engl J Med 356: 2271–2281 17538086 10.1056/NEJMoa066838

[bib13] Kirchner GI, Jacobsen W, Deters M, Christians U, Nashan B, Winkler M, Vidal C, Kaever V, Sewing K, Manns MP (2001) Fast quantification method for sirolimus and its major metabolites. Transplant Proc 33: 1091–1092 11267205 10.1016/s0041-1345(00)02430-1

[bib14] Lee KH, Ko BH, Paik JY, Jung KH, Choe YS, Choi Y, Kim BT (2005) Effects of anesthetic agents and fasting duration on 18F-FDG biodistribution and insulin levels in tumour-bearing mice. J Nucl Med 46: 1531–1536 16157537

[bib15] Loening AM, Gambhir SS (2003) AMIDE: a free software tool for multimodality medical image analysis. Mol Imaging 2: 131–137 14649056 10.1162/15353500200303133

[bib16] McSheehy PM, Allegrini PR, Ametamey S, Becquet M, Honer M, Schubiger PA, Lane H, O'Reilly T (2005) The anticancer agent RAD001 rapidly inhibits 18F-FDG uptake by sensitive but not resistant tumours. 52nd Meeting Society Nuclear Medicine

[bib17] Neshat MS, Mellinghoff IK, Tran C, Stiles B, Thomas G, Petersen R, Frost P, Gibbons JJ, Wu H, Sawyers CL (2001) Enhanced sensitivity of PTEN-deficient tumours to inhibition of FRAP/mTOR. Proc Natl Acad Sci USA 98: 10314–10319 11504908 10.1073/pnas.171076798 PMC56958

[bib18] Pugachev A, Ruan S, Carlin S, Larson SM, Campa J, Ling CC, Humm JL (2005) Dependence of FDG uptake on tumour microenvironment. Int J Radiat Oncol Biol Phys 62: 545–553 15890599 10.1016/j.ijrobp.2005.02.009

[bib19] Stroobants S, Goeminne J, Seegers M, Dimitrijevic S, Dupont P, Nuyts J, Martens M, van den BB, Cole P, Sciot R, Dumez H, Silberman S, Mortelmans L, van OA (2003) 18FDG-positron emission tomography for the early prediction of response in advanced soft tissue sarcoma treated with imatinib mesylate (Glivec). Eur J Cancer 39: 2012–2020 12957455 10.1016/s0959-8049(03)00073-x

[bib20] Tabernero J, Rojo F, Calvo E, Burris H, Judson I, Hazell K, Martinelli E, Cajal SR, Jones S, Vidal L, Shand N, Macarulla T, Ramos FJ, Dimitrijevic S, Zoellner U, Tang P, Stumm M, Lane HA, Lebwohl D, Baselga J (2008) Dose- and schedule-dependent inhibition of the mammalian target of rapamycin pathway with everolimus: a phase I tumour pharmacodynamic study in patients with advanced solid tumours. J Clin Oncol 26: 1603–1610 18332469 10.1200/JCO.2007.14.5482

[bib21] Tai YC, Ruangma A, Rowland D, Siegel S, Newport DF, Chow PL, Laforest R (2005) Performance evaluation of the microPET focus: a third-generation microPET scanner dedicated to animal imaging. J Nucl Med 46: 455–463 15750159

[bib22] Therasse P, Arbuck SG, Eisenhauer EA, Wanders J, Kaplan RS, Rubinstein L, Verweij J, Van Glabbeke M, van Oosterom AT, Christian MC, Gwyther SG (2000) New guidelines to evaluate the response to treatment in solid tumours. J Natl Cancer Inst 92: 205–216 10655437 10.1093/jnci/92.3.205

[bib23] Thomas GV, Tran C, Mellinghoff IK, Welsbie DS, Chan E, Fueger B, Czernin J, Sawyers CL (2006) Hypoxia-inducible factor determines sensitivity to inhibitors of mTOR in kidney cancer. Nat Med 12: 122–127 16341243 10.1038/nm1337

[bib24] Wei LH, Su H, Hildebrandt IJ, Phelps ME, Czernin J, Weber WA (2008) Changes in tumour metabolism as readout for mammalian target of rapamycin kinase inhibition by rapamycin in glioblastoma. Clin Cancer Res 14: 3416–3426 18519772 10.1158/1078-0432.CCR-07-1824

[bib25] Zasadny KR, Tatsumi M, Wahl RL (2003) FDG metabolism and uptake *vs* blood flow in women with untreated primary breast cancers. Eur J Nucl Med Mol Imaging 30: 274–280 12552346 10.1007/s00259-002-1022-z

